# Different SNP combinations in the GCH1 gene and use of labor analgesia

**DOI:** 10.1186/1744-8069-6-41

**Published:** 2010-07-15

**Authors:** Fatimah Dabo, Alfhild Grönbladh, Fred Nyberg, Inger Sundström-Poromaa, Helena Åkerud

**Affiliations:** 1 Department of Women's and Children's health, Uppsala University, Uppsala, Sweden; 2 Department of Pharmaceutical Biosciences, Division of Biological Research on Drug Dependence, Uppsala University, Uppsala, Sweden

## Abstract

**Background:**

The aim of this study was to investigate if there is an association between different SNP combinations in the guanosine triphosphate cyclohydrolase (GCH1) gene and a number of pain behavior related outcomes during labor. A population-based sample of pregnant women (n = 814) was recruited at gestational week 18. A plasma sample was collected from each subject. Genotyping was performed and three single nucleotide polymorphisms (SNP) previously defined as a pain-protective SNP combination of GCH1 were used.

**Results:**

Homozygous carriers of the pain-protective SNP combination of GCH1 arrived to the delivery ward with a more advanced stage of cervical dilation compared to heterozygous carriers and non-carriers. However, homozygous carriers more often used second line labor analgesia compared to the others.

**Conclusion:**

The pain-protective SNP combination of GCH1 may be of importance in the limited number of homozygous carriers during the initial dilation of cervix but upon arrival at the delivery unit these women are more inclined to use second line labor analgesia.

## Introduction

Labor is considered to be one of the most painful events in human experience. The perceived pain during labor is the result of a number of complex interactions involving physiological mechanisms, i.e. type of onset and duration of labor and the size of the fetus, as well as psychological mechanisms such as previous pain experiences and support [1-3].

From clinical practice it is well-known that women experience varying degrees of pain in labor, in turn raising the possibility that genetic predisposition may be of importance for pain perception. Indeed, a number of clinical studies have identified polymorphisms at several gene loci that are associated with differential sensitivity to experimental pain [4] and inbred strains of mice also display altered pain responses in models of neuropathic and inflammatory pain [5-7]. These studies strongly suggest that genetic factors play an important role underlying mechanisms of experience of pain. Although research within this field is limited in the obstetric setting, genetic variability of the μ-opioid receptor has recently been associated with intrathecal fentanyl analgesia requirements in laboring women [8].

It has recently been reported by Tegeder and colleagues that specific SNPs in the guanosine triphosphate cyclohydrolase (GCH1) gene are associated with reduced pain sensitivity in humans [9]. GCH1 is the rate limiting enzyme in the biosynthesis of 6(R)-L-erythro-5,6,7,8-tetrahydrobiopterin (BH4) [10,11]. BH4, in turn, is an essential cofactor in the synthesis of many pain modulators including catecholamines, serotonin and nitric oxide [12] and feed-forward activation via phenylalanine and feedback inhibition through BH4 regulates the activity of GCH1 [13]. The identified pain-protective SNP combination of GCH1 is composed of 15 SNPs found at different locations on the gene [9]. Recently, screening for the combination of three SNPs has been shown to be sufficient for definition of the pain-protective haplotype with high sensitivity and specificity: c.-9610G > A, c343+8900A > T, and c.*4279 > G [14].

Genetic polymorphisms that are of importance for regulation of pain during labor are scarcely studied and the importance of the pain-protective SNP combination of GCH1 in laboring women is unknown. We hypothesized that if genetic variability plays a clinically relevant role in pain perception during labor, the pain-protective SNP combination of GCH1 would be associated with altered use of labor analgesia and other pain behaviors in the ordinary, routine care of laboring women.

The aim of this study was to investigate if there is an association between the pain-protective SNP combination of GCH1 and a number of pain behavior related outcomes during labor.

## Results

### Background characteristics

Among all eligible women, 814 (approximately 65%) accepted to participate in the study. Genotyping was completed in 811 subjects. Of the 811 women with complete genotyping, 31 (3.8%) women delivered elsewhere, 53 (6.5%) were delivered by planned caesarean section, and 22 (2.7%) women were delivered by acute caesarean section before onset of labor. Three subjects who delivered before gestational week 30 and one subject with intrauterine fetal death were excluded. Consequently, 701 women entered active phase of labor and among these 676 were of Caucasian origin. Analyses were conducted in Caucasians only.

### Genotyping of the pain-protective SNP combination of GCH1

Among the 676 Caucasian subjects who had complete data on the SNP analyses in the study population, 15 (2.2%) were homozygous carriers, 180 (26.6%) heterozygous carriers and 481 (71.1%) were non-carriers of the pain-protective SNP combination of GCH1.

Demographic data and clinical variables for homozygous carriers, heterozygous carriers and non-carriers of the pain-protective SNP combination of GCH1 are given in Table 1. There were no major differences in sociodemographic and clinical variables between groups. Pain behavior related outcomes for homozygous carriers, heterozygous carriers and non-carriers of the pain-protective SNP combination of GCH1 are given in Table 2. Homozygous carriers of the GCH1 pain-protective SNP combination arrived in the delivery ward with a more advanced stage of cervical dilation than subjects heterozygous for the pain-protective SNP combination and non-carriers did (F(2,591) = 3.42; p = 0.033, adjusted for parity).

**Table 1:** Demographic data and clinical characteristics of the study population, according to GCH1 pain protecting haplotypes.

	Non-carriers (n = 481)	Heterozygous (n = 180)	Homozygous (n = 15)
Age, years	30.67 ± 4.9	31.0 ± 4.6	31.4 ± 5.1
BMI at first antenatal visit, kg/m^2^	24.2 ± 4.0	24.6 ± 4.3	22.3 ± 2.5
Pre-pregnancy smokers, n (%)^a^	52 (11.0%)	23 (13.1%)	3 (21.4%)
Married/cohabiting, n (%)	457 (95.0%)	169 (93.8%)	15 (100%)
Nulliparity, n (%)	221 (45.9%)	72 (40.0%)	10 (66.7%)
Singleton pregnancies	476 (99.0%)	180 (100%)	15 (100%)
			
Spontaneous start of delivery	411 (85.4%)	156 (86.7%)	13 (86.7%)
Gestational week	39.4 ± 1.5	39.3 ± 1.7	39.9 ± 1.3
Duration of labor, hours	7.4 ± 7.2	6.5 ± 5.7	6.5 ± 4.7
Use of oxytocin	219 (45.5%)	76 (42.2%)	7 (46.7%)
			
Vaginal delivery	405 (84.3%)	157 (87.2%)	12 (80.0%)
Vacuum extraction	53 (11.0%)	12 (6.7%)	1 (6.7%)
Caesarean section	23 (4.8%)	11 (6.1%)	2 (13.3%)

^a ^missing data in 13 subjects

**Table 2:** Labor pain behavior related outcomes according to GCH1 haplotypes

		Non-carriers (n = 481)	Heterozygous (n = 180)	Homozygous (n = 15)
Cervical dilation at arrival to the delivery unit^a^, cm		4.3 ± 2.5	4.1 ± 2.3	5.7 ± 2.2*
Cervical dilation at request of epidural analgesia^b^, cm		5.8 ± 1.9	5.3 ± 2.0	6.3 ± 2.3
Use of labor analgesia, n (%)	No use	44 (9.1%)	17 (9.4%)	1 (6.7%)
	Nitrous oxide	396 (82.3%)	143 (79.4%)	12 (80.0%)
	Acupuncture	155 (32.2%)	62 (34.4%)	6 (40.0%)
	Epidural analgesia	159 (33.1%)	52 (28.9%)	7 (46.7%)
	Second line analgesia	249 (51.8%)	92 (51.1%)	11 (73.3%)

*p = 0.033 in comparison to heterozygous carriers and non-carriers of the GCH1 haplotype, ANOVA adjusted for parity.

^a ^Data missing in 81 subjects

^b ^Data based on 159 non-carriers, 52 heterozygous and 7 homozygous carriers of the GCH1 haplotype.

### GCH1 and use of Labor Analgesia

A possible association between the pain-protective SNP combination of GCH1 and use of second line analgesia was suggested by the bivariate analysis (p = 0.10). This possible association was further analyzed with adjustment for possible confounders in a multivariate logistic regression model.

Factors associated with use of second line labor analgesia are given in Table 3. In the bivariate analyses nulliparity, induced labor, slight cervical dilation upon arrival in the delivery unit, and duration of labor were associated with second line labor analgesia. Independent explanatory factors for use of second line labor analgesia use were nulliparity, < 2 cm dilation of cervix at arrival in the delivery unit and duration of labor for more than 2 hours. Homozygous carriers of the pain-protective SNP combination had an increased risk of using second line labor analgesia, whereas heterozygous carriers did not differ in this aspect from non-carriers.

**Table 3:** Factors associated with use of second line labor analgesia

		Use of second line labor analgesia	Unadjusted OR	95% CI	Adjusted OR	95% CI
Parity	Parous	130 (34.9%)	1		1	
	Nulliparous	222 (73.3%)	5.12***	3.67 - 7.14	2.68***	1.73 - 4.16
Height			0.98	0.96 - 1.00	0.98	0.96 - 1.00
Start of labor	Spontaneous	291 (50.2%)	1		1	
	Induced	61 (63.5%)	1.73*	1.11 - 2.70	0.63	0.32 - 1.23
Cervical dilation at arrival to the delivery unit, cm^a^	> 5	83 (36.9%)	1		1	0.79 - 0.97
	2-4	178 (56.9%)	2.26***	1.59 - 3.20	1.34	0.85 - 2.13
	0-1	45 (78.9%)	6.42***	3.21 - 12.82	2.46*	1.05 - 5.76
Duration of labor^b^	0-2 hours	32 (17.6%)	1		1	
	2-5 hours	74 (45.1%)	3.85***	2.36 - 6.29	3.33***	1.92 - 5.80
	5-10 hours	109 (66.1%)	9.12***	5.54 - 15.03	7.13***	3.87 - 13.13
	> 10 hours	134 (87.0%)	31.41***	17.14 - 57.53	16.53***	7.68 - 35.62
GCH1 pain protecting genotype	Absent	249 (51.8%)	1		1	
	Heterozygous	92 (51.1%)	0.97	0.69 - 137	1.25	0.80 - 1.97
	Homozygous	11 (73.3%)	2.56	0.80 - 8.16	5.11*	1.09 - 23.96

^a ^Missing cases 81

^b ^Missing cases 11

81 subjects lacked data on cervical dilation at arrival to the delivery department. However, if cervical dilation was removed from the multivariate analysis, the odds ratio for homozygous carriers' use of second line labor analgesia was unchanged (OR 4.59, 95% CI 1.07 - 19.73).

## Discussion

Even though carriers of the pain-protective SNP combination of GCH1 in previous studies have been shown to be less sensitive to pain, the major finding of the present study was that this specific pain-protective SNP combination did not dramatically change pain perception or behavior during labor. Heterozygous carriers of the pain-protective SNP combination of GCH1 did not differ in any aspect from non-carriers in the possible pain behavior related outcomes of the study. Homozygous carriers of the pain-protective SNP combination of GCH1 comprised only 2.2% of the population-based sample but appeared to arrive in the delivery ward with a more advanced stage of cervical dilation than heterozygous and non-carriers did. This finding possibly indicates an increased tolerance to pain in the early stages of labor. However, once they arrived at the delivery ward, women with the pain-protective SNP combination were more inclined to use second line labor analgesia compared with heterozygous carriers and non-carriers.

The literature on the pain-protective SNP combination of GCH1 is conflicting and the pain-protective effect appears to be most evident in patients with neuropathic rather than nociceptive pain. Tegeder and colleagues [9] suggested that the pain-protective SNP combination is associated with less pain following discectomy for persistent radicular low back pain and their results have later been reproduced both by themselves [14] and by others [15]. Further studies on the pain-protective SNP combination of GCH1 have been more discouraging. Kim and colleagues [16] found no association between rated pain severity after surgical removal of molar teeth and the pain-protective SNP combination of GCH1, and similar negative findings were obtained in patients with chronic pancreatitis [17]. Presumably, the negative results in our study could be due to the fact that labor pain is mainly nociceptive or that pain modulating pathways affected by BH4 are not the primary ones during labor [12].

Pain during the first stage of labor is nociceptive and caused by distension of the lower uterine segment, dilation of cervix and the uterine muscle contractions. A number of neurotransmitters and chemical mediators are involved in the signaling, modulation, and perception of pain including bradykinin, catecholamines, serotonin, substance P, and nitric oxide [1,18]. In the second phase of labor the pain is also mainly nociceptive and arises from pressure on the vagina, vulva and perineum, mediated through the pudendal nerve. However, neuropathic pain from direct pressure on the lumbosaccral plexus is also of importance in this phase [18].

Another reason for the firm lack of results in our study could be due to the fairly blunt measures of pain perception and pain behavior that were used. If more detailed and specified protocols for labor analgesia had been used it is possible that more subtle differences between the different SNP combinations, and especially among the homozygous carriers, could have been revealed. However, even though our measures of pain severity were blunt they serve a purpose. If genetic variability plays a clinically relevant role in pain perception during labor that would justify pre-labor testing and counseling, the pain-protective genotype or haplotype of interest needs to prove itself in the ordinary, routine care of laboring women.

An increasing number of studies strongly suggest that genetic predisposition plays an important role in pain mechanisms [4], and although research is limited within the obstetric setting, it is plausible that certain pain-protective genotypes may contribute to the varying degree of perceived pain and/or pain behavior during labor. Recently homozygous carriers of the pain-protective 304G/G allele of the μ-opioid receptor were shown to require significantly less fentanyl for labor analgesia but also requested analgesia at a more advanced stage of cervical dilation [8].

Given that we only found signs of altered pain sensitivity among homozygous carriers of the pain-protective SNP combination of GCH1, the major limitation of our study is the number of women included. However, the results among the heterozygous carriers must be considered to be fairly robust. On the other hand, one of the strengths of the study is the homogenous population-based sample where Caucasians dominate. There is a known ethnic difference in pain sensitivity and genotype frequencies of the pain-protective SNP combination of GCH1 [16]. Kim and co-workers has shown that a homogenous population of a cohort is needed to avoid population stratification [16].

## Conclusions

In conclusion, our study indicates that the pain-protective SNP combination of GCH1 may be of importance in a limited number of homozygous carriers during the initial dilation of cervix but upon arrival at the delivery unit these women are more inclined to use second line labor analgesia. Clearly, presence of the pain-protective SNP combination of GCH1 does not contribute substantially to our understanding of labor pain and has little to offer in terms of individual counseling on labor analgesia.

## Methods

### Study population

Between March 1, 2007 and May 31, 2007 all women (age > 18 years) attending the second trimester routine ultrasound screening at Uppsala University Hospital were approached for study participation. In Sweden, all pregnant women are invited to an ultrasound examination at 16-18 weeks of gestation for estimation of the date of childbirth and approximately 97 percent of the Swedish pregnant population participate [19]. In Uppsala County, all routine ultrasound examinations are performed at Uppsala University Hospital which also is the only available delivery ward within the county. Hence, the study subjects represent a population-based sample.

Exclusion criteria for the study were (1) detection of malformation or missed abortion at the ultrasound examination, (2) inability to read and understand the study information because of language difficulties, and (3) not providing informed consent.

A blood sample was collected after the routine ultrasound examination in participating subjects.

Three months after delivery, the medical records of the women were thoroughly reviewed. The labor pain behavior related outcomes that were assessed in the study were 1) no use of analgesia during labor, 2) use of epidural analgesia during labor, 3) use of any type of second line analgesia, 4) stage of cervical dilation at arrival to the delivery unit, and 5) stage of cervical dilation at request of epidural analgesia. Second line labor analgesia was defined as any use of more than one type of analgesia during labor. Use of pudendus block (PDB) was not assessed as it was impossible from the medical records to distinguish whether it was used during or after delivery. As these outcomes are influenced by a number of confounders, demographic data together with a number of obstetric and delivery variables were also retrieved. Only cases with complete medical records, live births and those who had entered active phase of labor were included in the study.

The participating women gave written informed consent and the study was approved by the Independent Ethical Review Board at Uppsala University, Sweden.

### Sample collection

Blood samples were collected in tubes containing EDTA. After collection the samples were put immediately on ice, where they were kept for no longer than 2 h before they were centrifuged for 10 minutes at 5000 rpm. Plasma and buffy coat were separated and collected. The samples were then stored at -70°C until analyzed.

### DNA isolation

Total genomic DNA for the SNP genotyping assay was extracted from whole blood using the Magtration 12GC system (Precision System Science, Chiba, Japan) and the Magazorb^® ^DNA Common Kit-200 (Precision System Science, Chiba, Japan) as described earlier [20]. From each sample 200 μl whole blood was used and the final volume of the DNA extract was 100 μl. The concentration of the DNA was determined with Nanodrop Spectrophotometer (Nanodrop Technologies Inc., Wilmington, DE, USA).

### SNP genotyping

Three SNPs defined by Tegeder et al [14] as a pain-protective haplotype of GCH1 were used. The SNPs analyzed were c.-9610G > A (dbSNP rs8007267G > A), c343+8900A > T (dbSNP rs3783641A > T) and c.*4279 > G (dbSNP rs10483639C > G). The SNPs were determined by TaqMan SNP genotyping assay (assay numbers C_25800745, C_1545138 and C_3044867 respectively, Applied Biosystems, Foster City, CA, USA). Briefly, the assay included target-specific PCR primers and TaqMan MGB probes labeled with two special dyes, FAM and VIC. Applied Biosystems designed the primers and allele-specific probes. To each well in a 384-well plate, genomic DNA (5 ng), water, TaqMan Universal PCR master mix and TaqMan genotyping assay mix was added, in a total volume of 5ul. The genotyping was carried out, according to the manufacturers' instructions, using the ABI7900HT genetic detection system (Applied Biosystems) with the following amplification protocol: 10 min at 95°C and 40 cycles of 15 s at 92°C and 1 min at 60°C.

### Genotyping analyses

Genotyping was completed on 811 subjects. Among them the numbers of homozygous carriers, heterozygous carriers and non-carriers of the 3 SNPs used were in accord with the Hardy-Weinberg equilibrium (c.-9610G > A: *x*^2 ^= 0.22, p = 0.65; c343+8900A > T: *x*^2 ^= 0.35, p = 0.55; c.*4279 > G: *x*^2 ^= 0.0005, p = 0.98). The genotype frequencies for c.-9610G > A were GG 67.6%, AG 28.9%, and AA 3.4%, for c343+8900A > T were AA 66.4%, AT 29.8%, and TT 3.8% and for c.*4279 > G AA 65.3%, AT 31.0%, and TT 3.7% respectively.

### Statistical analyses

The study had a power of 80% to detect a difference in 1 cm on cervical dilation at arrival to the delivery unit with 500 women in the group of non-carriers compared to 200 women in the group of carriers (heterozygous and homozygous) with an α-value of 0.05.

Demographic and obstetric variables were compared between homozygous, heterozygous and non-carriers of the pain-protective SNP combination by one-way analysis of variance (ANOVA) and Tukey HSD or logistic regression (with non-carriers as reference). Any significant differences between groups were adjusted for relevant confounders by analysis of covariance (ANCOVA) or multivariate logistic regression.

Multivariate logistic regression analyses were used to calculate unadjusted and adjusted odds ratios for use of second line labour analgesia. The following maternal factors were analyzed: maternal age as completed years at the time of the delivery, parity as primipara or not, marital status as married/cohabiting with a partner or other marital status. Pre-pregnancy smoking, recorded at the first visit to antenatal care, was categorized into non-smoking (not daily smoking), and smoking (one or more cigarettes per day). The first trimester height and weight were used to calculate body mass index (BMI), kg/m^2^. Pregnancy and delivery data were categorized as follows: gestational age according to the result of the second trimester ultrasound screening, birth weight in grams, start of labor as spontaneous or induced, duration of labor as time from arrival in the delivery unit to parturition (categorized into quartiles), cervical dilation at arrival in the delivery unit (categorized as 0-1 cm, 2-4 cm and more than 5 cm), oxytocin use (yes/no). Only variables with significant bivariate association with use of second line analgesia (p < 0.25) were included in the final multivariate model. Parous subjects were significantly older than nulliparous subjects (p < 0.001), and as parity was a stronger explanatory variable it was used in the final model. Likewise, induced onset of labor and use of oxytocin were significantly related (*x*^2 ^= 61.6, p < 0.001) and for this reason, only spontaneous onset of labor was used in the final model. A two-sided p-value less than 0.05 was considered as significant.

## Competing interests

The authors declare that they have no competing interests.

## Authors' contributions

FD designed the study, collected samples, analyzed the data and drafted the manuscript. AG did the SNP analyses. FN designed the study and drafted the manuscript. I S-P and HÅ designed and coordinated the study, analyzed the data, and finished the final draft of the manuscript. All authors read and approved the final manuscript.
